# Lifelong high-fat, high-sucrose diet causes sex-specific heart dysfunction in mouse offspring

**DOI:** 10.20935/acadmed7821

**Published:** 2025-07-24

**Authors:** Yun-Ju Fang, Filip Konecny, Eunhee Chung

**Affiliations:** 1 Department of Kinesiology, University of Texas at San Antonio, San Antonio, TX, USA.; 2 Department of Surgery, McMaster University, Hamilton, ON, Canada.; 3 Department of Comparative Medicine, Moffitt, Tampa, FL, USA.

**Keywords:** maternal diet, post-weaning diet, obesity, offspring, hemodynamics, pressure–volume loop

## Abstract

Maternal obesity and high-fat, high-sucrose (HFHS) diets during development increase cardiometabolic risk in offspring, but long-term, sex-specific cardiac effects remain underexplored. This study examined how continuous HFHS exposure impacts cardiac function in male and female mice. Female dams were fed a control standard chow (CON) diet or HFHS diet for 8 weeks before pregnancy, continuing through gestation and lactation. Offspring were maintained on their dam’s diet until 29–32 weeks of age. Body composition and cardiac function were assessed using pressure–volume (P–V) loop analysis. HFHS offspring exhibited increased body weight and fat mass, with males showing greater adiposity. Lean mass was higher in males, but relative lean mass decreased in both sexes by 22 weeks in response to the HFHS diet. Cardiac assessments revealed load-dependent and load-independent impairments. HFHS exposure increased end-diastolic and end-systolic volumes, reduced ejection fraction, and lowered end-systolic elastance, indicating systolic dysfunction in both sexes. Diastolic function showed sex-specific alterations; HFHS exposure in males led to slower myocardial relaxation (less negative dP/dt min), while in females it increased end-diastolic elastance (E*ed*), suggesting greater ventricular stiffness. Ventricular–arterial coupling (E*es*/E*a*) was reduced in HFHS-exposed animals of both sexes, with females showing more pronounced impairments. Our results highlight sex-specific cardiac dysfunction in HFHS-exposed offspring, with females more susceptible to myocardial stiffness and coupling deficits. This underscores the need for sex-tailored interventions to mitigate long-term cardiovascular risks from early-life HFHS exposure.

## 1. Introduction

Maternal obesity and the consumption of energy-dense diets, particularly those high in fat and sucrose (HFHS), are increasingly prevalent and have been linked to long-term cardiometabolic consequences in offspring [1, 2]. In preclinical models, maternal obesogenic diets induce fetal programming effects that are not easily reversible. Even when offspring are weaned onto a control diet, those born to obese mothers often exhibit increased body weight, insulin resistance, and adverse cardiac remodeling later in life [3–6]. Moreover, continued exposure to obesogenic diets after weaning has been shown to further exacerbate cardiometabolic dysfunction [7–9]. As a result, the sex-specific impact of lifelong HFHS exposure—including preconception, gestation, lactation, and post-weaning periods—on cardiac function remains poorly understood. While our study design does not allow the separation of prenatal versus postnatal dietary effects, it was intentionally designed to model continuous maternal and postnatal HFHS exposure, providing insight into the cumulative impact on adult cardiac function in both male and female offspring.

Sex differences play a critical role in the development of diet-induced cardiovascular dysfunction [10–12]. Despite growing recognition of these effects, most prior studies have focused predominantly on male offspring due to their greater susceptibility to obesity and insulin resistance [3, 4, 8, 9]. Rodent studies have demonstrated that males exposed to high-fat diets often develop more severe cardiac remodeling, including left ventricular hypertrophy and both systolic and diastolic dysfunction [13]. Conversely, females often exhibit preserved cardiac function [10, 12], though subtle diastolic impairments with clinical implications may still occur [14]. While estrogen is thought to confer cardio-protective effects, chronic exposure to HFHS diets may blunt this protection, particularly by exacerbating diastolic dysfunction [14]. For example, female offspring from high-fat-fed dams have been reported to show elevated end-diastolic pressure and reduced ventricular compliance compared to males [15]. HFHS diets have also been shown to impair endothelial function through oxidative stress and reduced nitric oxide signaling, further contributing to cardiovascular risk in both preclinical models and humans [9].

Despite these observations, few studies have systematically examined the combined effects of maternal and lifelong HFHS exposure on intrinsic myocardial function in both sexes using sensitive hemodynamic assessments. Pressure–volume (P–V) loop analysis offers a sensitive approach to detect subclinical changes in contractility, relaxation, and ventricular–arterial coupling—parameters that standard echocardiography may overlook [16–18]. In this study, we investigated the effects of maternal and continuous HFHS diet exposure on body composition and cardiac function in male and female offspring. We hypothesized that prolonged HFHS exposure would exacerbate adiposity and impair cardiac performance in a sex-specific manner. We tested this hypothesis using a mouse model, where dams and their offspring were fed either a control standard chow (CON) diet or an HFHS diet. Outcomes were evaluated at 29–32 weeks of age through body composition analysis and hemodynamic measurements derived from P–V loop data. This approach offers valuable new insights into the long-term, sex-specific cardiovascular effects of early-life and sustained dietary stress.

## 2. Materials and methods

### 2.1. Experimental design and animal model

**Figure 1 •:**
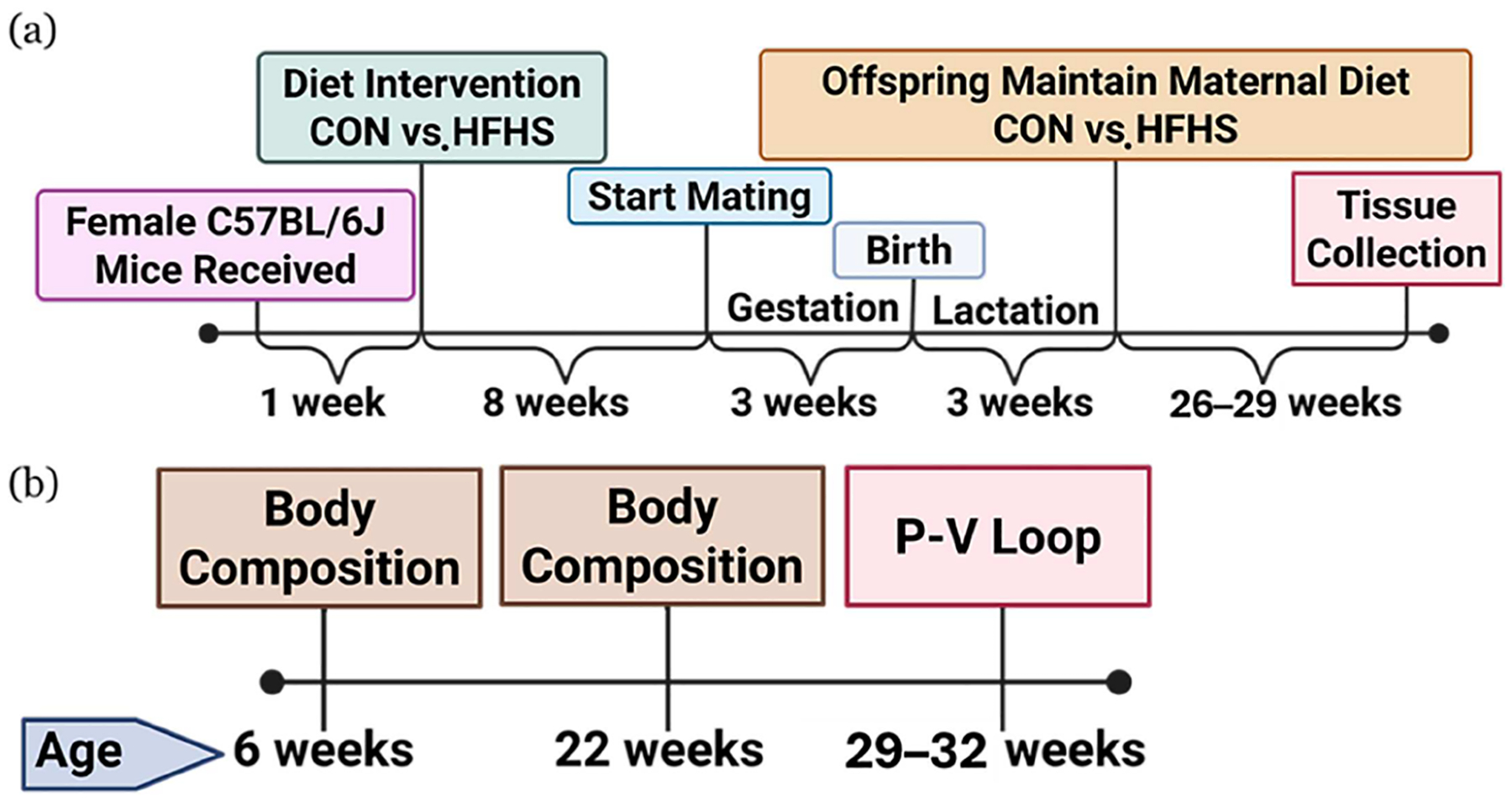
Schematic study design. Timeline of (**a**) maternal and (**b**) offspring diet interventions, and body composition assessments and cardiac function analysis using pressure–volume (P–V) loop measurements. CON—control standard chow diet; HFHS—high-fat, high-sucrose diet.

Virgin female C57BL/6J mice (5 weeks old; Jackson Laboratory, Bar Harbor, ME, USA) were housed in accordance with the University of Texas at San Antonio IACUC protocol (#MU109, approved 2023 Jul 1). All experimental procedures and reporting adhered to the ARRIVE (Animal Research: Reporting of In Vivo Experiments) guidelines [19]. After a 7-day acclimation on a CON diet (15% fat, 21% protein, 64% carbohydrate; 3.42 kcal/g), females were randomly assigned at 6 weeks of age to either the CON diet or a HFHS diet (45% fat, 40% carbohydrate with 34% sucrose; 4.7 kcal/g; Inotiv, Madison, WI, USA) for 8 weeks before mating. Mating was conducted using a harem breeding system to minimize paternal effects, as described previously [20]. Briefly, female mice remained on their assigned diets, except during mating, when they were switched to the control diet to match the males, who were fed a CON diet. This approach was used to minimize paternal dietary influences on offspring development as reported previously [21]. Maternal body weight was monitored weekly until weaning. Due to significantly lower pup survival in offspring born to HFHS dams [22, 23], litter size normalization was not possible. To minimize potential litter size effects, litters from CON dams (which ranged from 5 to 11 pups) were culled to five pups on postnatal day 10, while all pups from HFHS dams were retained to account for higher postnatal mortality. Offspring were weaned on postnatal day 21, at which time body weight was recorded. They remained on their respective maternal diets until euthanasia at 29–32 weeks of age. To account for maternal influences and reduce litter bias [24], one male and female mouse per litter was selected, and if the pups were within same litter, the average was reported. A schematic overview of the maternal and offspring study timeline, including dietary interventions and data collection points, is shown in Figure 1.

### 2.2. Body composition

Fat mass and lean mass were assessed in conscious mice at 6 and 22 weeks of age using time-domain nuclear magnetic resonance (Minispec LF50H; Bruker, Billerica, MA, USA), following previously described protocols [25]. Mice were placed in clear plastic tubes with a plunger to restrict movement, and measurements were completed within approximately two minutes without anesthesia. This method allows for repeated, noninvasive assessments of body composition with high precision and minimal stress. Body composition data were used to track longitudinal changes in response to continuous dietary exposure from early life to adulthood.

### 2.3. Hemodynamic measurements

Stroke volume (SV) was calculated from the LVOT diameter and velocity–time integral, as described previously [26]. The SV was used to calibrate volume measurements. P–V loop measurements were performed in animals aged 29–32 weeks under isoflurane anesthesia using a 1.2 F admittance catheter (Transonic Systems Inc., Ithaca, NY, USA) and the data were analyzed using LabChart Pro and LabScribe software. Hemodynamic parameters included heart rate (HR), ejection fraction (%EF), dP/dt max and min, SV, stroke work (SW), and arterial elastance (E*a*). Load-independent indices, including end-systolic elastance (E*es*), end-diastolic elastance (E*ed*), preload recruitable stroke work (PRSW), and ventricular–arterial coupling (E*es*/E*a*), were derived from transient inferior vena cava (IVC) occlusion, following previously validated protocols [16, 27].

### 2.4. Statistical analysis

Statistical analyses were conducted using GraphPad Prism (version 10). Maternal body weight was compared using an unpaired t-test. Two-way ANOVA was used to assess the main effects of sex, diet, and their interaction. Post hoc comparisons were restricted to predefined, biologically relevant contrasts: male CON vs. male HFHS, female CON vs. female HFHS, male CON vs. female CON, and male HFHS vs. female HFHS. Fisher’s Least Significant Difference (LSD) test was used for these planned comparisons to enhance sensitivity while limiting the total number of comparisons. Results are reported as mean ± standard error of the mean (SEM), and statistical significance was defined as *p* < 0.05.

## 3. Results

### 3.1. Maternal body weight

Maternal body weight (BW) was monitored at multiple time points to evaluate the effects of dietary intervention before and during pregnancy and lactation (Table 1). Initial body weights did not differ between groups prior to dietary assignment. However, dams fed the HFHS diet exhibited significantly higher BW prior to mating (after 8 weeks of dietary intervention) and at weaning compared to CON-fed dams (*p* < 0.05). No significant difference was observed between groups on postnatal day 10. These findings confirm that the HFHS diet induced a maternal obese phenotype prior to conception and maintained elevated BW through lactation.

### 3.2. Offspring body weight

**Figure 2 •:**
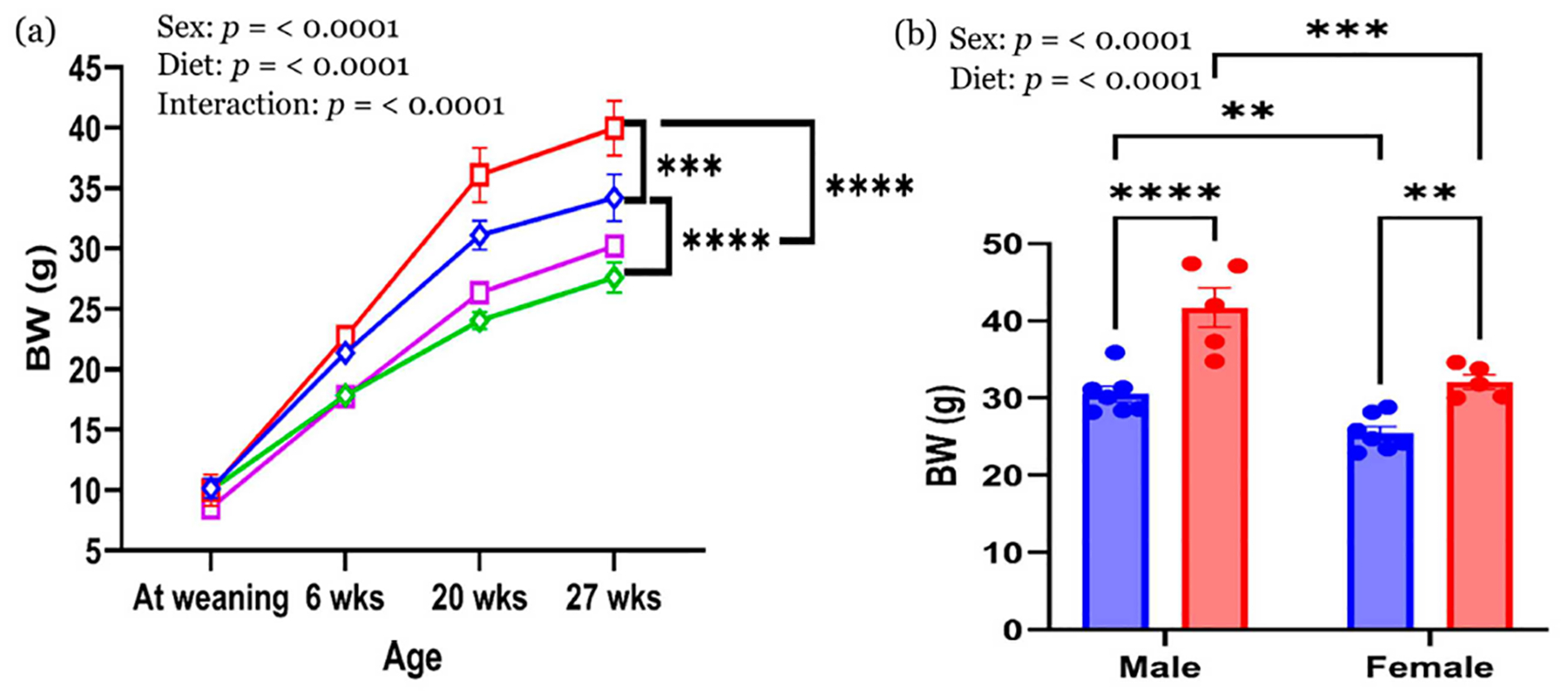
Body weight. (**a**) BW trajectories from weaning, 6, 20, and 27 weeks of age and (**b**) final BW at 29–32 weeks. Values are expressed as mean ± with the standard error of the mean (SEM). ** *p* < 0.01; *** *p* < 0.001; **** *p* < 0.0001. *n* = 7–9 in CON male; *n* = 5 in HFHS male; *n* = 7–11 in CON female; *n* = 5–6 in HFHS female. BW—body weight; CON—control standard chow diet; HFHS—high-fat, high-sucrose diet; blue rhombus—CON male; red square—HFHS male; green rhombus—CON female; pink square—HFHS female; blue circle—CON group; red circle—HFHS group.

BW trajectories from weaning to 27 weeks are shown in Figure 2a. At weaning and 6 weeks of age, no significant differences in BW were observed between CON and HFHS groups within each sex. By 20 and 27 weeks, significant main effects of both sex and diet were evident. Males weighed more than females regardless of diet, and HFHS-fed offspring of both sexes had higher BW than their CON-fed counterparts. Final BW at 29–32 weeks is summarized in Figure 2b. Male HFHS offspring had the highest BW, followed by male CON, female HFHS, and female CON offspring. Post hoc analysis revealed significant differences between HFHS and CON groups within each sex (*p* < 0.01) and between males and females within each diet group (*p* < 0.01). These results confirm additive effects of sex and chronic HFHS diet exposure on offspring body weight.

### 3.3. Body composition

**Figure 3 •:**
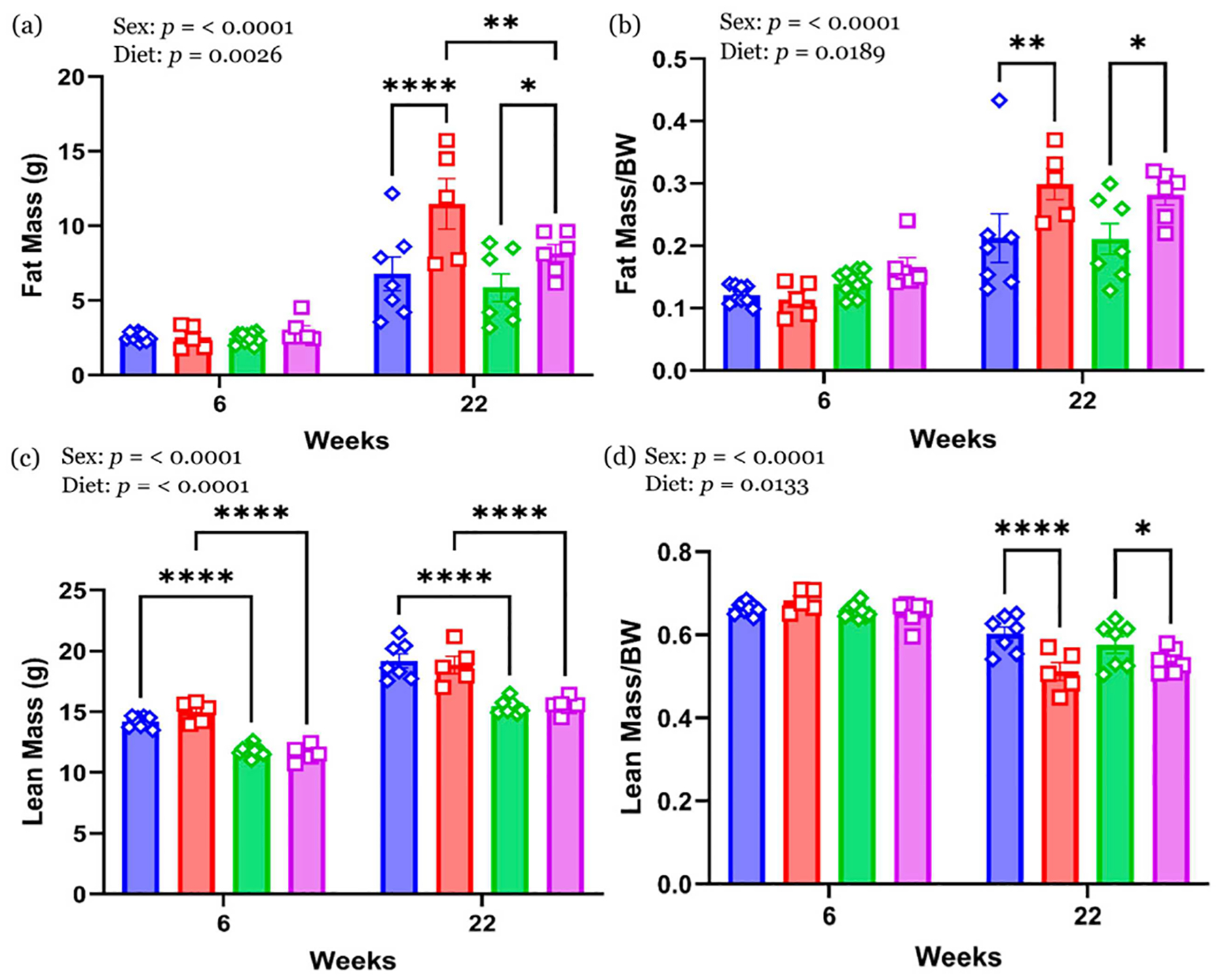
Body composition at 6 and 22 weeks. (**a**) Fat mass and (**b**) fat mass/BW. (**c**) Lean mass and (**d**) lean mass/BW. Values are expressed as mean ± with the standard error mean (SEM). *n* = 7–9 in CON male; *n* = 5 in HFHS male; *n* = 7–11 in CON female; *n* = 6 in HFHS female. * *p* < 0.05; ** *p* < 0.01; **** *p* < 0.0001. BW—body weight; CON—control standard chow diet; HFHS—high-fat, high-sucrose diet; blue rhombus—CON male; red square—HFHS male; green rhombus—CON female; pink square—HFHS female.

No significant differences in absolute fat mass or fat mass/BW were observed at 6 weeks across diet groups or sexes (Figure 3a,b). However, by 22 weeks, both male and female HFHS offspring exhibited significantly greater fat mass and fat mass/BW compared to their CON-fed counterparts (*p* < 0.05). Male HFHS offspring had the highest absolute fat mass among all groups. Lean mass (Figure 3c) was significantly higher in males than females at both time points, regardless of diet. No significant differences in lean mass/BW were observed at 6 weeks (Figure 3d). At 22 weeks, HFHS-fed animals of both sexes had significantly lower lean mass/BW compared to CON-fed animals (*p* < 0.05), indicating a diet-induced shift in body composition toward increased adiposity (Figure 3d).

### 3.4. Heart weight

**Figure 4 •:**
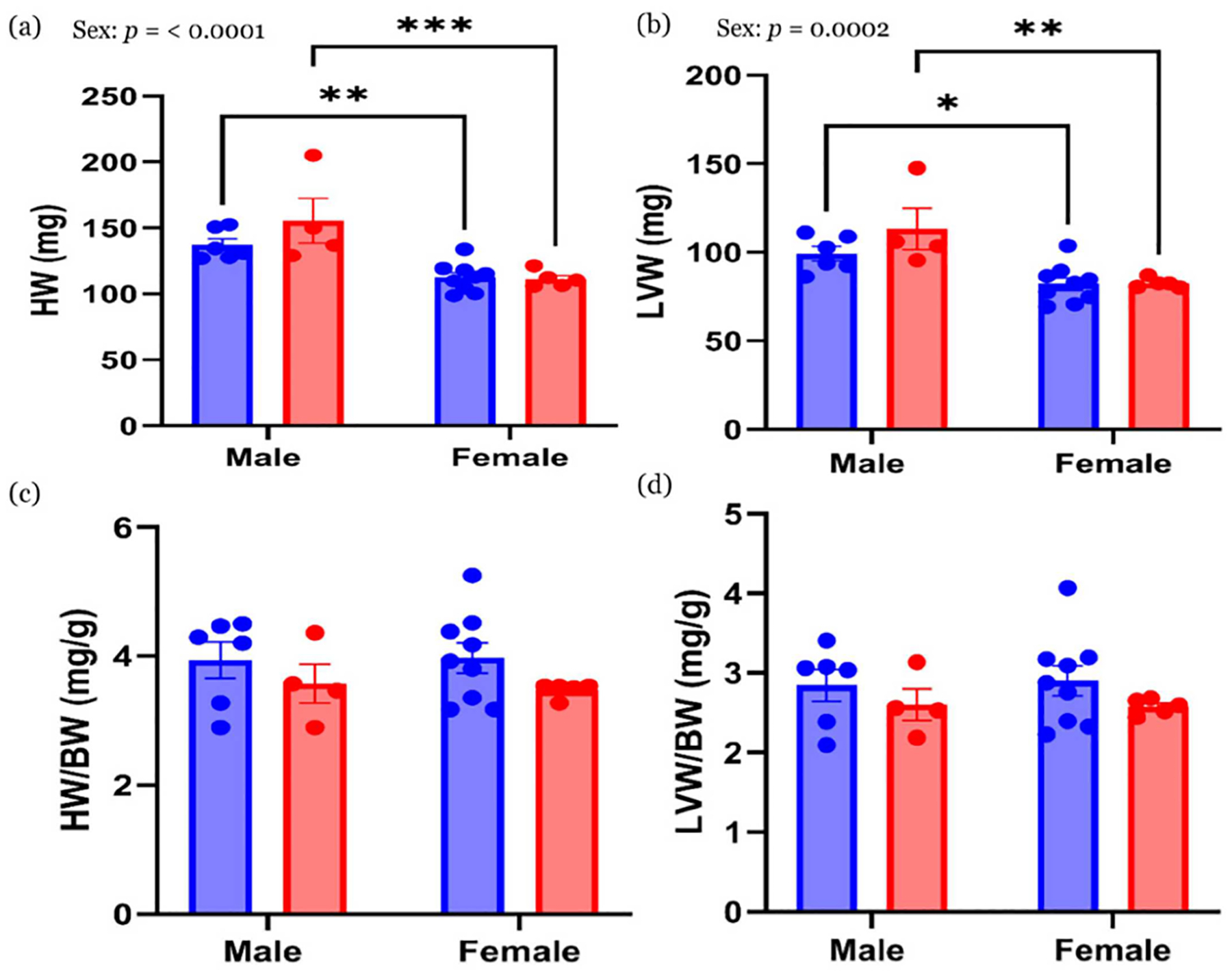
Heart morphology. (**a**) Absolute HW and (**b**) LVW. (**c**) HW/BW and (**d**) LVW/BW. Values are expressed as mean ± with the standard error mean (SEM). *n* = 6 in CON male; *n* = 4 in HFHS male; *n* = 9 in CON female; *n* = 5 in HFHS female. * *p* < 0.05; ** *p* < 0.01; *** *p* < 0.001. HW—heart weight; LVW—left ventricle weight; BW—body weight; CON—control standard chow diet; HFHS—high-fat, high-sucrose diet; blue circle—CON group; red circle—HFHS group.

Absolute heart weight (HW) and left ventricle weight (LVW) (Figure 4a,b) were significantly higher in males than females, regardless of diet (*p* < 0.05). When normalized to BW (HW/BW and LVW/BW), no significant differences were observed between groups (Figure 4c,d).

### 3.5. Cardiac function

#### 3.5.1. Load-dependent cardiac performance

Hemodynamic parameters derived from load-dependent indices of systolic and diastolic function are summarized in Tables 2 and 3. HR, E*a*, and dP/dt max did not differ significantly across sex or diet groups. For systolic function, SV was significantly greater in males than females, with no significant effect of diet. In contrast, end-systolic volume (ESV) was significantly elevated in HFHS animals compared to CON-fed animals across both sexes, indicating impaired systolic emptying. Consistent with this, %EF was significantly lower in HFHS animals. SW was significantly higher in males than females, but SW normalized to BW (SW/BW) did not differ significantly among groups. End-systolic pressure (ESP) was significantly higher in males than females, with no main effect of diet or interaction.

For diastolic function, end-diastolic volume (EDV) was significantly increased in HFHS animals compared to CON animals, particularly in males. End-diastolic pressure (EDP) did not differ significantly. dP/dt min, a measure of diastolic relaxation, was significantly impaired in male HFHS animals compared to male CON animals, indicating slowed relaxation. The ratio of dP/dt max to −dP/dt min was significantly increased in HFHS animals compared to CON animals. Tau, the isovolumic relaxation constant, did not differ significantly across groups.

#### 3.5.2. Transient preload-independent cardiac performance

**Figure 5 •:**
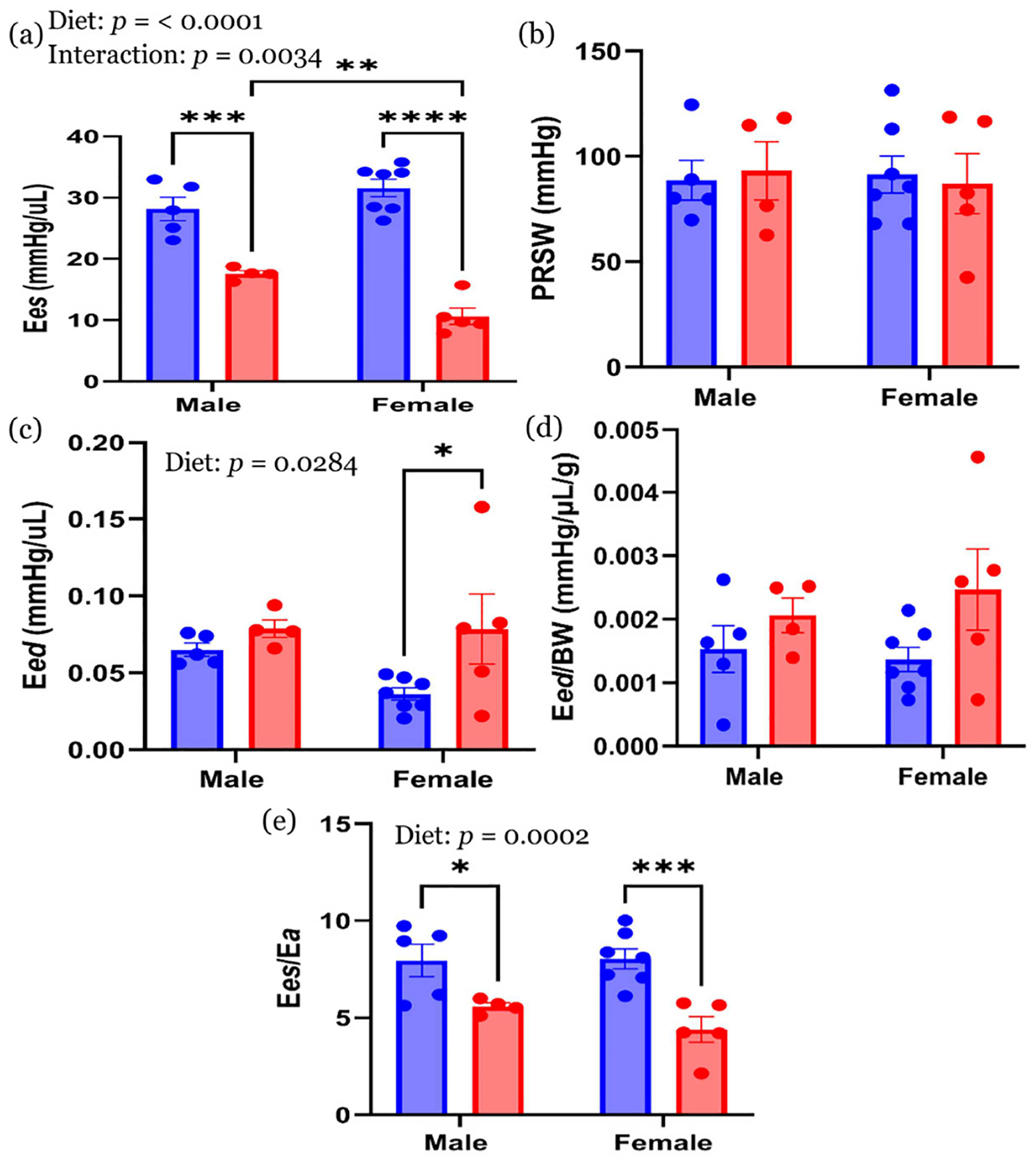
Load-independent indices of cardiac performance. (**a**) E*es*, (**b**) PRSW, (**c**) E*ed*, (**d**) E*ed*/BW, and (**e**) E*es*/E*a*. Values are expressed as mean ± with the standard error mean (SEM). *n* = 5 in CON male; *n* = 4 in HFHS male; *n* = 7 in CON female; *n* = 5 in HFHS female. * *p* < 0.05; ** *p* < 0.01; *** *p* < 0.001; **** *p* < 0.0001. E*es*—end-systolic elastance; PRSW—preload recruitable stroke work; E*ed*—end-diastolic elastance; BW—body weight; E*es*/E*a*—ventricular–arterial coupling; CON—control standard chow diet; HFHS—high-fat, high-sucrose diet; blue circle—CON group; red circle—HFHS group.

Transient preload-independent cardiac performance was assessed during inferior vena cava (IVC) occlusion (Figure 5). E*es*, representing the ESPVR slope, was significantly lower in HFHS animals than in CON for both sexes (Figure 5a). E*es* was also significantly lower in female HFHS animals compared to male HFHS animals (*p* < 0.01). PRSW did not differ significantly for both variables, i.e., diet or sex (Figure 5b). For diastolic performance, E*ed* was significantly elevated in female HFHS animals compared to female CON animals (*p* < 0.05) (Figure 5c). When normalized to BW (Figure 5d), E*ed*/BW did not differ significantly across groups. E*es*/E*a* was significantly reduced in HFHS animals compared to CON in both sexes (Figure 5e).

## 4. Discussion

This study highlights critical sex-specific cardiovascular effects of continuous HFHS diet exposure from gestation through late adulthood in mouse offspring. Using P–V loop analysis—a gold-standard method for assessing intrinsic myocardial function [27]—subclinical cardiac dysfunction was detected, which standard echocardiography could not identify. The study’s novelty lies in its life-course approach, mirroring sustained obesogenic dietary patterns in humans [28], and its comprehensive assessment of both load-dependent and load-independent cardiac parameters in male and female offspring.

Maternal body weight data confirmed that HFHS exposure induced modest but significant pre-pregnancy obesity, aligning with prior models of maternal overnutrition and metabolic stress [22, 23, 29]. BW convergence during lactation—also observed in previous studies—is likely attributable to increased caloric demand and intake in CON dams [29]. These maternal adaptations are relevant because maternal metabolic health directly influences the fetal environment and downstream cardiometabolic programming [23, 30].

Lifelong HFHS exposure led to shared systolic impairments in both sexes, as evidenced by reduced E*es* and %EF, along with diminished E*es*/E*a*. Diastolic abnormalities, however, were sex-specific; male offspring exhibited impaired relaxation (reduced -dP/dt min), while female offspring showed increased myocardial stiffness (elevated E*ed*). These divergent phenotypes suggest distinct underlying pathophysiological mechanisms, with implications for sex-specific cardiovascular risk trajectories. These results build upon prior studies demonstrating that maternal HFHS diets predispose offspring to cardiac dysfunction [8, 15], including systolic impairment in male offspring [3, 8], and diastolic dysfunction in female offspring [3, 8, 15, 31, 32] even when offspring are weaned to control diets and followed into late adulthood. Our continuous exposure model magnifies these impairments, capturing both shared and sex-specific vulnerabilities [28].

Notably, prolonged HFHS intake in our study compromised contractility (reduced E*es*) and ventricular–arterial coupling (reduced E*es*/E*a*) in both sexes, aligning with studies showing that HFHS diets impair calcium handling and exacerbate systolic dysfunction [33]. The sex-specific divergence in diastolic function—specifically elevated E*ed* in females—suggests a loss of estrogen-mediated cardioprotection. Estrogen typically enhances nitric oxide availability and reduces vascular stiffness through ER*α* signaling [34], but prolonged HFHS diets impair estradiol and other sex hormones [35]. These changes may contribute to increased myocardial collagen deposition and mitochondrial dysfunction, which preferentially impair ventricular compliance in females [36]. Such mechanisms align with clinical findings showing that women with metabolic syndrome are more susceptible to early-onset diastolic stiffness than men [13, 34]. In addition, maternal metabolic dysfunction promotes endothelial and hepatic inflammation, establishing a pro-inflammatory, pro-oxidant intrauterine environment [30]. This state alters placental gene expression and redox signaling in a sex-specific manner, shaping fetal immune programming and growth trajectories. Such prenatal conditions likely predispose the myocardium to long-term functional impairments. Systemic endothelial dysfunction commonly observed in HFHS models—including impaired nitric oxide signaling, oxidative stress, and vascular stiffening—may further reduce coronary perfusion and ventricular compliance, thereby amplifying intrinsic myocardial dysfunction [30, 34].

The shared ventricular–arterial uncoupling observed in both sexes suggests a systemic vascular contribution to HFHS-induced cardiac dysfunction [37]. However, this dysfunction occurred independently of cardiac hypertrophy. While some studies report hypertrophy in offspring of obese dams [4, 15], our results are consistent with others showing no such remodeling [8, 32]. Indeed, ventricular dilation, rather than hypertrophy, appears to underlie the observed dysfunction, as supported by increased EDV in both sexes. This pattern aligns with reports that hypertrophy in male offspring may resolve by 12 weeks, giving way to dilation and functional decline [3].

### 4.1. Limitations

Several limitations should be acknowledged. First, the study did not assess histological or molecular analyses of myocardial structure, oxidative stress, inflammation, or mitochondrial function that could clarify the mechanistic underpinnings of the observed cardiac changes [30]. Second, as diet exposure continued postnatally, it was not possible to disentangle prenatal from postnatal contributions to the cardiac phenotype. Lastly, the male HFHS group included only four animals due to litter size constraints and technical exclusions. Nevertheless, statistically significant impairments in E*es*, %EF, E*es*/E*a*, and dP/dt min were observed, suggesting a robust phenotype. Future studies integrating mechanistic investigations are also needed to assess myocardial fibrosis, vascular reactivity, mitochondrial efficiency, and inflammatory signaling. Particular attention should be paid to sex-specific hormonal and redox regulatory pathways, which may help explain the divergent diastolic responses observed in male and female offspring. Such work is essential for identifying critical windows of vulnerability and designing targeted prevention strategies for diet-induced cardiovascular disease.

## 5. Conclusions

This study investigated the long-term effects of continuous HFHS diet exposure from preconception through adulthood on cardiac function in male and female mouse offspring. Using high-fidelity P–V loop analysis, we identified sex-specific patterns of cardiac dysfunction. Both sexes exhibited systolic impairment and reduced ventricular–arterial coupling, while diastolic abnormalities differed by sex—impaired relaxation in males and increased myocardial stiffness in females. These findings occurred in the absence of significant cardiac hypertrophy and were instead associated with increased ventricular volumes. Together, the results demonstrate that lifelong HFHS exposure induces distinct and measurable alterations in cardiac function, shaped by biological sex.

## Figures and Tables

**Table 1 •:** Maternal body weight at key timepoints.

Body weight (BW)	Dams	
	CON (*n* = 13)	HFHS (*n* = 9)
Initial	16.5 ± 0.4	16.9 ± 0.4
Before Mating	20.2 ± 0.4	22.5 ± 0.6 *
PND 10	29.7 ± 0.4	28.4 ± 0.6
At weaning	28.2 ± 0.6	30.4 ± 0.4 *

* indicates significant differences between diet groups (*p* < 0.05), determined by unpaired *t*-test. Values are expressed as mean ± standard error of the mean (SEM). CON—control standard chow diet; HFHS—high-fat high-sucrose diet; PND 10—postnatal day 10.

**Table 2 •:** Load-dependent indices of systolic function.

Parameters	Male		Female		Two-way ANOVA		
	CON (*n* = 5)	HFHS (*n* = 4)	CON (*n* = 7)	HFHS (*n* = 5)	Sex	Diet	Interaction
HR (bpm)	479 ± 8.7	435 ± 19.8	475 ± 16.6	492 ± 10.7	ns	ns	ns
SV (μl)	29.4^b^ ± 1.3	31.6^b^ ± 0.6	24.7^a^ ± 0.8	26.5^a^ ± 1.1	0.0002	ns	ns
ESV (μl)	7.3^y^ ± 0.9	16.5^x^ ± 1.5	11.7^y^ ± 1.2	16.4^x^ ± 1.0	ns	<0.0001	ns
%EF	56.3^y^ ± 2.0	43.0^x^ ± 0.9	49.3^y^ ±1.9	42.3^x^ ± 2.1	ns	<0.0001	ns
E*a* (mmHg μl)	3.5 ± 0.4	3.2 ± 0.2	3.4 ± 0.3	2.6 ± 0.3	ns	ns	ns
SW (mmHg μl)	2702.3^b^ ± 192.0	2683.2^b^ ± 135.5	1841.3^a^ ± 109.7	1909.9^a^ ± 323.4	0.0009	ns	ns
SW/BW (mmHg /g)	81.7 ± 8.5	69.6 ± 6.7	73.2 ± 5.6	63.8 ± 7.2	ns	ns	ns
ESP (mmHg)	91.8^b^ ± 4.5	85.0 ± 4.0	74.8^a^ ± 4.5	78.0 ± 7.3	0.0403	ns	ns
dP/dt max (mmHg/s)	7195.3 ± 625.7	6403.3 ± 1344.8	6876.6 ± 830.0	6747.7 ± 1146.2	ns	ns	ns

Different letters (^a^ and ^b^ for sex effect within diet groups; ^x^ and ^y^ for diet effect within sexes) are significantly different by two-way ANOVA (*p* < 0.05).

Values are expressed as mean ± with the standard error mean (SEM). HR—heart rate; SV—stroke volume; ESV—end-systolic volume; EF—ejection fraction; E*a*—arterial elastance; SW—stroke work; SW/BW—relative stroke work normalized body weight; ESP—end-systolic pressure; dP/dt max—maximal slope of systolic pressure increment; CON—control standard chow diet; HFHS—high-fat, high-sucrose diet; ns—not significant.

**Table 3 •:** Load-dependent indices of diastolic function.

Parameters	Male		Female		Two-way ANOVA		
	CON (*n* = 5)	HFHS (*n* = 4)	CON (*n* = 7)	HFHS (*n* = 5)	Sex	Diet	Interaction
EDV (μl)	36.7^y^ ± 1.6	48.0^bx^ ± 2.0	36.4 ± 1.6	40.0^a^ ± 1.7	0.0309	0.0006	0.0427
EDP (mmHg)	7.1 ± 2.6	4.0 ± 1.2	2.8 ± 1.2	4.9 ± 1.6	ns	ns	ns
dP/dt min (mmHg/s)	−8741.1^y^ ± 594.9	−5619.1^x^ ± 1510.9	−7006.5 ± 529.5	−5931.4 ± 839.7	ns	0.0229	ns
Ratio (dP/dt max/-dP/dt min)	0.9^y^ ± 0.03	1.2^x^ ± 0.10	1.0^y^ ± 0.06	1.2^x^ ± 0.06	ns	0.0013	ns
T*au* (ms)	7.6 ± 0.8	8.8 ± 0.8	7.9 ± 0.5	9.3 ± 1.0	ns	ns	ns

Different letters (^a^ and ^b^ for sex effect within diet groups; ^x^ and ^y^ for diet effect within sexes) are significantly different by Two-way ANOVA (*p* < 0.05).

Values are expressed as mean ± with the standard error mean (SEM). EDV—end-diastolic volume; EDP—end-diastolic pressure; dP/dt min—minimal slope of diastolic pressure decrement; T*au*—isovolumic relaxation constant; CON—control standard chow diet; HFHS—high-fat, high-sucrose diet; ns—not significant.

## Data Availability

All data supporting the findings of this publication are available within this article.
